# Somatotype and body composition of healthy adult men and women and their contribution to civilization diseases risk

**DOI:** 10.1186/s40101-025-00420-8

**Published:** 2026-01-09

**Authors:** Lipowicz Anna, Pol Aleksandra, Świerczyńska Karolina, Nierwińska Katarzyna, Nowak Zbigniew, Myśliwiec Andrzej, Knapik Andrzej

**Affiliations:** 1 Department of Anthropology, Wrocław University of Environmental and Life Sciences, Wrocław, Poland; 2 Pohl, Victus Med, Rybnik, Poland; 3 Laboratory of Physiotherapy and Physioprevention, Institute of Physiotherapy and Health Sciences, Academy of Physical Education in Katowice, Katowice, Poland; 4 Department of Adapted Physical Activity and Sport, Faculty of Health Sciences in Katowice, Medical University of Silesia, Katowice, Poland

**Keywords:** Heath-Carter algorithms, Sheldon, BIA, Endomorphy, Mesomorphy, Ectomorphy

## Abstract

**Background:**

Somatotypes describe changes in body structure and allow estimations of biological differences and similarities between subjects. They can also highlight the relationship between body composition and risk factors for worsening health.

**Objectives:**

The study aimed to evaluate lifestyle disease risk in adult men and women based on resting blood pressure and body composition. Somatotyping was used to determine body type as endomorphic, mesomorphic, or ectomorphic.

**Methods:**

The study used the anthropological measurements of 344 subjects, 179 men and 165 women, to determine somatotype using the Sheldon method. Body composition analysis employed an electrical bioimpedance method, while a sphygmomanometer measured heart rate and systolic and diastolic blood pressure. The study has been explicitly described as a cross-sectional study involving 344 adults aged 18–75 years (mean = 40.8 years), including 179 men (18–73 years, mean = 40.3 years) and 165 women (18–75 years, mean = 41.4 years).

**Results:**

Men were more likely to be mesomorphic, and women were more likely to have an endomorphic somatotype. Individuals with a predominantly mesomorphic somatotype had higher total muscle mass. Furthermore, mesomorphs had the highest levels of body composition components (muscle, bone, fat-free mass, and water), and ectomorphs had the lowest. Generally, somatotype components correlated significantly with age, with a greater proportion of endomorphy and mesomorphy in older age groups. Body mass index (BMI) and waist circumference varied in women depending on somatotype. In men, BMI, waist circumference, and waist-to-hip ratio (WHR) were also somatotype dependent, with similarly elevated values found in endomorphs and mesomorphs and lower values in ectomorphs. Resting blood pressure was associated with somatotype in women, while ectomorphy was negatively associated with risk factors for disease in older men.

**Conclusions:**

Somatotype may provide complementary information to individual measurements for assessing biological risk and predisposition to disease among adults, especially women. As such, somatotype can be considered a useful and appropriate tool for describing health-related characteristics across different populations, including both healthy and diseased individuals.

## Introduction

Somatotyping allows for a numerical description of body build, with the Sheldon method, modified by Heath–Carter, most commonly used to express somatotype in a three-number classification based on three components of body constitution. The method describes body types as endomorphic (relative fatness), mesomorphic (musculoskeletal robustness relative to body height), and ectomorphic (relative linearity) [1].

Somatotype is an important physical indicator for assessing the efficiency and physical and motor abilities of athletes [2, 3]. In most sporting disciplines, the top-ranking athletes are more mesomorphic and endomorphic than the rest of the population [1]. Individuals with an ectomorphic build are predisposed to endurance sports, while individuals characterized by an endo-mesomorphic component are better at sports requiring strength or power [1]. However, using somatotypes to assess health status or predict health deterioration (i.e., general health, cardiorespiratory fitness, disease risk, overall morbidity, and mortality) is much less common.

Body composition analysis involves quantifying various components of the human body (e.g., body fat, muscle and/or bone mass [BM], and total body water [TBW]) and is a known determinant of biological condition and health status in all age groups [4, 5]. Individuals with excessive body weight and visceral fat have a higher risk of cardiovascular and metabolic diseases [6, 7], while lower muscle mass reduces life expectancy and increases the risk of death from various causes [8]. In turn, excessively low body weight is a risk factor for poor condition and health and lowers the chances of survival for people suffering from diseases [9].

Body composition assessment is a valuable method of estimating nutritional health by measuring elements of body composition, such as fat-free mass (FFM), fat mass, and TBW [10]. However, in addition to environmental factors, genetics significantly influence body fat levels and distribution [11].

Somatotyping and body composition analysis have advantages and disadvantages depending on the purpose of their application and the characteristics of the group assessed. While the relationship between body build and body composition is known in the case of athletes [12–14], sick individuals [15], and children [16], the somatotype of healthy adults not involved in any sports training is rarely analyzed. Such analysis could be useful for assessing health, lifestyle, diet, and willingness to undertake healthy leisure activities. Therefore, the present study aimed to evaluate the body build and composition of adult men and women using the Heath–Carter somatotype method. In addition, somatotypes were characterized by taking into account body composition and risk factors for health decline.

## Material and methods

The study measured the data of 344 adults (18–75 years [mean = 40.8]), including 179 men (18–73 years [mean = 40.3 years]) and 165 women (18–75 years [mean = 41.4 years]), as part of the “SMART—activity monitoring and training rationalization system” (UDA-RPSL.01.02.00–24.00-045E/19-00) project. The Bioethics Committee KBE (8/2020) approved the project. Participant recruitment was conducted through social media and among people familiar with the research team to find volunteers who were required to present a medical certificate confirming good health and the absence of chronic disease symptoms. Individuals with diagnosed chronic conditions were excluded. Although such recruitment produced a heterogeneous sample, it reflects real-world, community-based conditions and was consistent with the study aim of investigating somatotypes in a non-preselected adult population. The International Physical Activity Questionnaire-Short Form (IPAQ-SF) and Global Physical Activity Questionnaire (GPAQ) determined the level of subjects’ physical activity [17]. A weekly energy expenditure of less than 600 metabolic equivalent minutes (MET-mins) per week was used as an inclusion criterion to identify adults with low or very low levels of physical activity.

Anthropological measurements were taken using the methods described by Martin and Saller [18] and included body height, body weight, fat skinfold thickness (triceps skinfold measured on the posterior surface of the arm above the triceps brachii muscle, subscapular skinfold measured directly below the inferior angle of the scapula, and suprailiac skinfold measured in a lateral line above the hip plate), breadth of the elbow epiphysis (humerus), breadth of the knee epiphysis (femur), mid-arm circumference at rest and under tension, and circumference of the lower leg at the greatest bulge of the gastrocnemius muscle. Other measurements included body mass index (BMI) and waist-to-hip ratio. Somatotype was determined for each subject using the Heath–Carter modification of the Sheldon method.

Regression equations were used to calculate the individual somatotype components [1].

The endomorphy component was obtained using the algorithm as follows:

$$\mathrm{Endomorphy}\:=\:-0.7182\:+\:0.1452\times\sum\mathrm{endosum}-0.00068\times\sum\mathrm{endosum}^2\:+\:0.0000014\times\sum\mathrm{endosum}^3$$

where:

$$\mathrm{endosum}\hspace{0.17em}=\hspace{0.17em}\sum\text{fat skinfolds x}(\frac{170.18}{height}).$$

The mesomorphy component was obtained using the algorithm as follows:

$$\mathrm{Mesomorphy}\:=\:0.85\:\times\:\text{humerus breadth}\:+\:0.601\:\times\:\text{femur breadth}\:+\:0.188\:\times\text{corrected arm girth}\:+\:0.161\:\times\text{corrected calf girth}-0.131\:\times\:height\:+\:4.5$$

For the ectomorphy component, a HWR (height–weight ratio) calculation was first performed, and, depending on its value, the corresponding formula was used:

$$HWR\:=\frac{height\;\left[cm\right]}{\sqrt[3]{weight\;\left[kg\right]}}$$

If HWR is greater than or equal to 40.75, then ectomorphy = 0.732 × HWR – 28.58.

If HWR is less than 40.75 but greater than 38.25, then ectomorphy = 0.463 × HWR – 17.63.

If HWR is equal to or less than 38.25, then ectomorphy = 0.1

When analyzing the values of the somatotype components, 1 of 13 somatotypes was assigned to each subject [1] and then stratified into three categories according to the dominant type (endomorph, mesomorph, or ectomorph).

Electrical bioimpedance is determined body composition in each subject using a Tanita instrument (TANITA MC-780 P MA BK). For body composition assessment, body fat percentage (FATP), skeletal muscle mass (SMM), BM, FFM (muscle, bone, water, and inner organ mass), and TBW were selected.

Systolic and diastolic blood pressure and heart rate were measured in the sitting position after a 5-min rest using an upper arm sphygmomanometer. Measurements were repeated twice, and the mean value was recorded, following procedures commonly applied in exercise testing protocols [19].

The data obtained were analyzed using Statistica 13.1. Since most of the biological characteristics studied did not have normal distributions, nonparametric Kruskall-Wallis tests and the Mann–Whitney *U*-test assessed the significance of differences between men and women and between variables characterizing individuals with different somatotypes. Dunn’s post hoc tests with Bonferroni correction were performed to avoid the problem of multiple comparisons. Meanwhile, Spearman correlation R analysis assessed the association between somatotypes and age, and chi-squared tests estimated the frequency of each somatotype. Outliers were identified a priori using the ±3 standard deviation (SD) criterion within sex-specific distributions. As a sensitivity check, the analyses were also verified using Tukey’s interquartile range (IQR) rule; the results remained unchanged after excluding outlying observations.

## Results

Men and women differed significantly in body height, body weight, triceps fat skinfold thickness, elbow and knee epiphysis breadth, and arm and lower leg circumference (Table 1). Men had higher values in all of the aforementioned body measurements, except for the thickness of the arm skinfold, where women achieved higher values (Table 1). Men also had higher TMM, BM, FFM (muscle, bone, water, and internal organ mass), and TBW. Women, on the other hand, were characterized by higher % body fat FATP (Table 1).

**Table 1:** Body build of adult men and women

	Men			Women			
	Mean ± SD	± 95%CI	min–max	Mean ± SD	± 95%CI	min–max	Z/p
Height [cm]	179.1 ± 6.6	179.1–180.1	162.0–194.5	164.1 ± 6.1	163.1–165.0	147.5–180.0	14.68***
Weight [kg]	83.4 ± 13.9	81.4–85.5	57.5–136.7	65.4 ± 11.3	63.6–67.1	40.0–118.9	11.52***
Triceps skinfold [mm]	12.1 ± 7.1	11.1–13.1	3.0–73.0	20.5 ± 6.9	19.4–21.6	5.0–40.5	−11.52***
Subscapular skinfold [mm]	17.8 ± 8.5	16.5–19.0	6.5–4.0	19.1 ± 9.6	17.6–20.6	5.5–58.0	−1.17
Suprailiac skinfold [mm]	21.1 ± 11.9	19.4–22.9	4.5–85.0	22.5 ± 10.8	20.9–24.2	4.5–58.0	−1.65
Humerus breadth [mm]	7.0 ± 0.6	6.9–7.0	5.3–10.0	5.9 ± 0.5	5.8–6.0	5.0–8.5	13.84***
Femur breadth [mm]	9.8 ± 0.8	9.6–9.9	4.0–13.0	9.1 ± 0.8	8.9–9.2	7.8–12.0	9.37***
arm circ. at rest [cm]	31.7 ± 3.3	31.2–32.2	25.0–41.0	28.4 ± 3.6	27.8–28.9	20.3–44.0	8.81***
arm circ. under tension [cm]	34.9 ± 3.5	34.3–35.4	27.5–48.0	29.8 ± 3.4	29.3–30.3	22.0–45.0	11.65***
calf circ. [cm]	38.3 ± 3.2	37.8–38.7	30.0–48.5	36.5 ± 3.0	36.0–36.9	22.5–47.0	5.25***
FAT P [%]	20.1 ± 6.1	19.2–21.0	8.2–37.2	27.1 ± 7.0	26.0–28.1	10.7–48.6	−8.90***
TMM [kg]	62.7 ± 7.5	61.5–63.9	47.6–94.6	45.0 ± 4.9	44.2–45.7	31.4- 58.0	14.47***
BM[kg]	3.3 ± 0.4	3.3–3.4	2.5–4.8	2.4 ± 0.3	2.4–2.5	1.7–3.3	15.22***
FFM[kg]	66.0 ± 7.8	64.9–67.2	50.1–99.4	47.1 ± 5.2	46.3–47.9	33.1- 61.1	15.44***
TBW [kg]	46.3 ± 5.0	45.6–47.1	35.4–69.7	31.6 ± 2.8	31.1–32.0	24.4- 41.5	15.95***
Endomorphy	4.67 ± 1.74	4.4–4.9	1.27–10.16	5.95 ± 1.80	5.7–6.2	1.63–10.64	−6.52***
Mesomorphy	5.28 ± 1.54	5.0–5.5	1.42–10.79	4.54 ± 1.76	4.3–4.8	1.71–12.30	4.73***
Ectomorphy	1.78 ± 1.16	1.6–2.0	0.10–4.83	1.69 ± 1.19	1.5–1.9	0.10–4.73	0.68
Somatotype	4.67–5.28–1.78			5.95–4.54–1.69			

Legend: level of significance *** *p *< 0.001

**Fig. 1:**
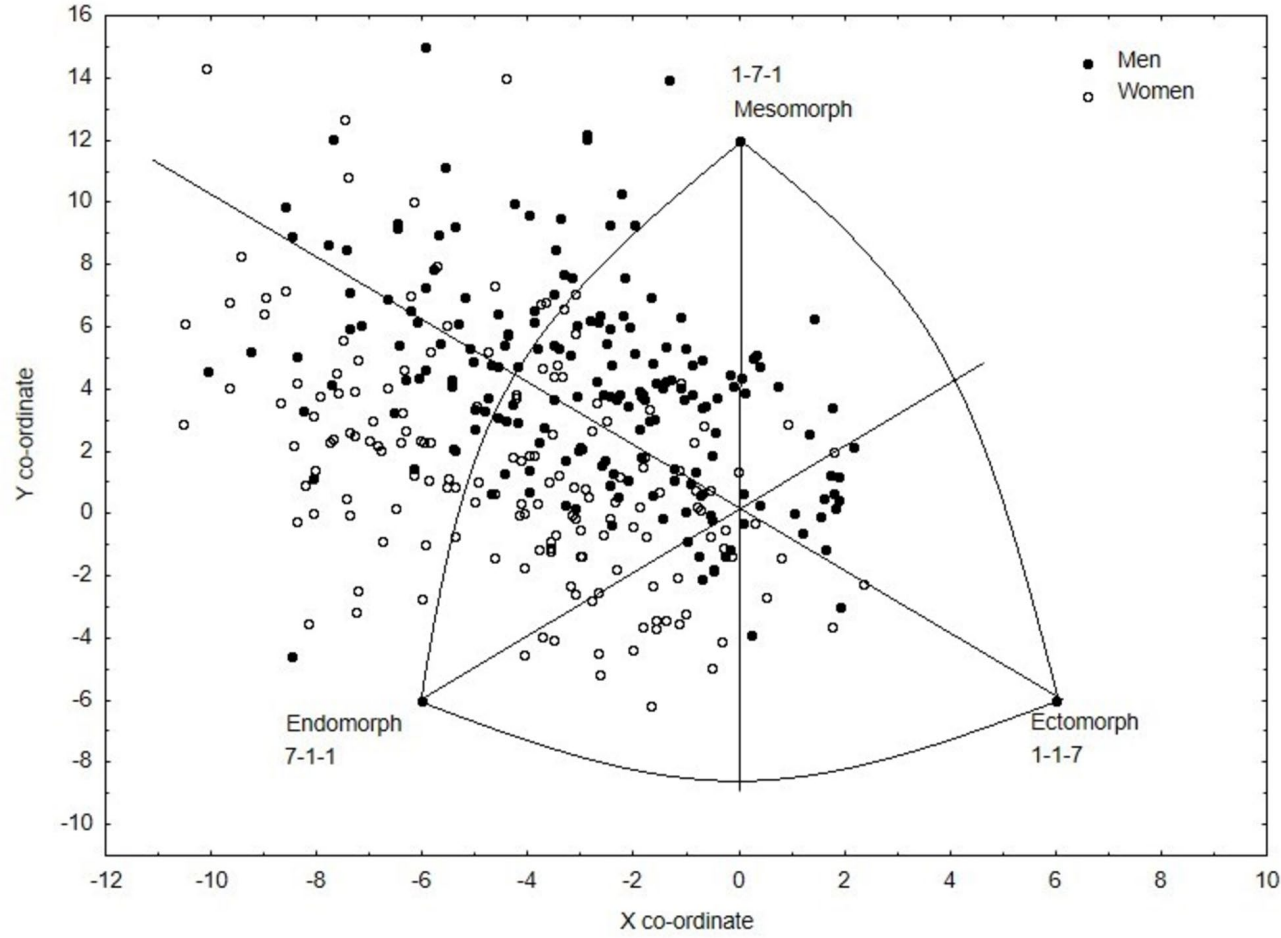
Somatograph of all participants

The somatotype of adult women can be described as 5.95–4.54–1.69 and the somatotype of men as 4.67–5.28–1.78, with women characterized by a larger endomorphic component than men (Z = −6.52, *p* < 0.001). An inverse relationship was shown for the mesomorphic component, with men achieving a higher proportion than women (*Z* = 4.73,* p* < 0.001). No statistically significant sex differences were found for the ectomorphy component (Table 1 and Fig. 1).

Somatotype components depended significantly on participant age. With age, the proportion of endomorphy and mesomorphy increased, while ectomorphy decreased in both sexes (Table 2). In addition, Spearman’s correlation coefficients were higher for women than men, indicating greater importance of age in shaping the female somatotype.

**Table 2:** Spearman correlation coefficients (R) between somatotype components and age in men and women

	**Men**	**Women**
Endomorphy	0.15*	0.34***
Mesomorphy	0.22**	0.44***
Ectomorphy	−0.27***	−0.44***

Legend: level of significance * *p* < 0.05; ** *p* < 0.01; *** *p* < 0.001

Significant correlations were found between somatotype components and age (Table 2). In men, endomorphy (R = 0.15, *p* < 0.05) and mesomorphy (R = 0.22, *p* < 0.01) were positively associated with age, while ectomorphy was negatively associated (R = −0.27, *p* < 0.001). In women, stronger correlations were observed: endomorphy (R = 0.34, *p* < 0.001) and mesomorphy (R = 0.44, *p* < 0.001) increased with age, whereas ectomorphy decreased (R = −0.44,* p* < 0.001).

A statistically significant difference was found in the frequency of the somatotypes due to participant sex (Table 3). Among men, the mesomorphic somatotype dominated, while the endomorphic somatotype was most frequently observed in women (*X*^2^ = 68.4; df = 2; *p* < 0.001).

**Table 3:** Number and frequency of men and women by somatotype

		Men	Women
Endomorph	% n exp.-obs	34.08 61 −37.3	77.58 128 37.3
Mesomorph	% n exp.-obs	58.66 105 33.7	19.39 32 −33.7
Ectomorph	% n exp.-obs	7.26 13 3.6	3.03 5 −3.6
	*X*^2^ = 68.4; df = 2; *p* < 0.001		

Legend: exp.-obs. – expected minus observed (residuals)

Somatotype significantly differentiated body composition in men and women (Table 4). Endomorphs had the highest fat component, while the mesomorphic type had higher skeletal muscle mass (SMM), bone mass (BM), FFM, and TBW. In men, endomorphs had, in addition to fat content, similar levels of other body composition components to mesomorphs, with frequent co-occurrence of both components. Ectomorphs significantly differed from endo- and mesomorphs; they had the lowest proportions of separate body composition components (lowest fatness, muscularity, BM, FFM, and TBW).

**Table 4:** Age and body composition of men and women with various somatotypes

	Endomorphs		Mesomorphs		Ectomorphs		Post hoc test			
	Mean ± SD	± 95%CI	Mean ± SD	± 95%CI	Mean ± SD	± 95%CI	Endo-Meso	Meso-Ecto	Endo-Ecto	H/p
**Women (*n* = 165)**										
Age [years]	41.6 ± 11.8	39.6–43.7	41.7 ± 11.9	37.4–46.0	35.8 ± 14.7	18.2–54.0	ns	ns	ns	0.7
% fat	27.9 ± 6.4	26.8–29.1	25.1 ± 8.4	22.1–28.1	19.3 ± 1.7	17.5–21.0	0.018	ns	0.003	17.0***
TMM [kg]	44.9 ± 4.7	44.0–45.7	46.4 ± 5.0	44.5–48.4	39.1 ± 5.0	32.8–45.3	ns	0.019	ns	7.6*
BM [kg]	2.4 ± 0.3	2.4–2.5	2.5 ± 0.3	2.4–2.6	2.1 ± 0.3	1.8–2.5	ns	0.036	ns	6.6*
FFM [kg]	47.0 ± 5.0	46.1–47.9	48.3 ± 5.3	46.4–50.3	41.2 ± 5.3	34.6–47.8	ns	0.036	ns	6.4*
TBW [kg]	31.5 ± 2.7	31.1–32.0	32.4 ± 2.9	31.3–33.4	27.5 ± 2.1	24.9–30.1	ns	0.001	0.007	12.4**
**Men (*n* = 179)**										
Age [years]	39.3 ± 12.4	36.2–42.5	41.4 ± 13.5	38.8–44.1	34.8 ± 12.6	27.2–42.4	ns	ns	ns	2.7
% fat	22.8 ± 5.3	21.5–24.2	19.3 ± 6.1	18.1–20.5	13.8 ± 3.2	11.9–15.7	< 0.001	0.003	< 0.001	34.5***
SMM [kg]	63.4 ± 5.7	61.8–64.9	63.6 ± 7.9	61.9–65.2	54.0 ± 5.6	50.6–57.3	ns	< 0.001	< 0.001	18.1***
BM [kg]	3.4 ± 0.4	3.3–3.5	3.4 ± 0.4	3.3–3.4	2.9 ± 0.3	2.7–3.0	ns	< 0.001	< 0.001	19.4***
FFM [kg]	66.9 ± 6.3	65.3–68.5	66.7 ± 8.1	65.1–68.3	56.8 ± 5.8	53.3–60.3	ns	0.001	0.001	18.1***
TBW	47.1 ± 4.2	46.0–48,2	46.7 ± 5.1	45.7–47.7	39.8 ± 3.1	37.9–41.6	ns	< 0.001	< 0.001	24.0***

Legend: ns – non significant, level of significance * *p* < 0.05; ** *p* < 0.01; *** *p *< 0.001

Among the risk factors for poor health, somatotype significantly differentiated BMI and waist circumference in women and BMI, waist circumference, and WHR in men (Table 5). Generally, ectomorphs had significantly the lowest values, while endomorphs and mesomorphs had similar values for these measures. In addition, somatotype differentiated between systolic and diastolic blood pressure, achieving significance in women but not men (Table 5). Ectomorphs had the lowest systolic and diastolic blood pressure values, while endomorphs of both sexes had the highest.

**Table 5:** Cardiovascular risk factors for men and women with various somatotypes

	Endomorphs		Mesomorphs		Ectomorphs		Post hoc test			
	Mean ± SD	± 95%CI	Mean ± SD	± 95%CI	Mean ± SD	± 95%CI	Endo-Meso	Meso-Ecto	Endo-Ecto	H/p
Women (*n* = 165)										
BMI [kg/m^2^]	24.3 ± 3.7	23.7–25.0	25.1 ± 5.2	23.2–27.0	18.5 ± 1.3	16.8–20.2	ns	0.001	0.001	13.2**
WHR	0.8 ± 0.2	0.8–0.8	0.8 ± 0.1	0.8–0.8	0.8 ± 0.1	0.7–0.9	ns	ns	ns	1.7
Waist circumference [cm]	80.5 ± 11.1	78.5–82.4	79.3 ± 11.9	75.0–83.6	67.5 ± 3.9	62.7–72.3	ns	0.028	0.005	10.2**
Heart rate in rest [beats]	83.6 ± 11.6	81.6–85.7	81.4 ± 9.2	78.0–84.8	81.6 ± 11.6	67.2–96.0	ns	ns	ns	0.8
Systolic BP in rest [mmHg]	122.0 ± 11.3	120.0–124.1	121.4 ± 14.9	115.7–127.0	106.0 ± 8.9	94.9–117.1	ns	ns	0.020	7.9*
Diastolic BP in rest [mmHg]	70.3 ± 8.5	68.8–71.9	66.9 ± 7.6	64.0–69.8	62.0 ± 4.5	56.4–67.6	ns	ns	0.095	8.3*
Men (*n* = 179)										
BMI [kg/m^2^]	26.5 ± 3.6	25.6–27.4	26.3 ± 3.8	25.6–27.1	20.4 ± 1.1	19.7–21.0	ns	ns	< 0.001	33.6***
WHR	0.9 ± 0.1	0.9–1.0	0.9 ± 0.1	0.9–0.9	0.9 ± 0.1	0.8–0.9	ns	ns	0.016	7.8*
Waist circumference [cm]	94.5 ± 10.7	91.7–97.2	91.3 ± 11.1	89.2–93.5	78.5 ± 5.5	75.2–81.8	ns	ns	< 0.001	26.6***
Heart rate in rest [beats]	82.4 ± 11.8	79.3–85.4	79.3 ± 11.9	76.9–81.8	79.6 ± 14.8	70.7–88.6	ns	ns	ns	2.1
Systolic BP in rest [mmHg]	128.7 ± 15.2	124.7–132.7	125.7 ± 12.4	123.1–128.4	123.5 ± 11.4	116.6–130.4	ns	ns	ns	1.4
Diastolic BP in rest [mmHg]	71.4 ± 8.7	69.1–73.7	68.7 ± 7.6	67.1–70.3	67.7 ± 7.3	63.3–72.1	ns	ns	ns	3.3

Legend: ns – non significant, level of significance * *p* < 0.05; ** *p *< 0.01; *** *p* < 0.001

## Discussion

An assessment of somatotype in adults with low levels of physical activity showed that men and women not only differ in size, proportion, and body composition [20] but also in somatotype. To some extent, these differences may be related to sex-related factors (genetics), dietary patterns, physical activity, and other sociocultural or environmental factors that directly affect variation within the population. In men, the mesomorphic and endomorphic components dominated to a similar degree, while the endomorphic component was most prevalent in women. Direct links between sex and fat or muscle dominance were suggested by a body composition study, where male endomorphs and mesomorphs had similar body composition and female endomorphs had significantly more body fat and less muscle mass than mesomorphic women. Mesomorphic women were also characterized by higher BM, FFM, and TBW than endomorphs and ectomorphs. In contrast, the body composition of male endomorphs did not differ from that of mesomorphs. Ectomorphic women had 6–8 kg less fat, 6–7 kg less skeletal muscle mass, 6–7 kg less FFM, and 4–5 kg less water compared to women defined as endomorphs and mesomorphs. Ectomorphic men averaged 5–9 kg less fat, 9–10 kg less skeletal muscle mass, 10 kg FFM, and 7 kg less water than endomorphs and mesomorphs.

Many studies have reported endomorphy as the dominant component for women, while mesomorphy in men is similar to endomorphy [21, 22]. Comparisons of male and female somatotypes from 10 countries clearly indicated sexual dimorphism for this trait [23, 24].

Sex differences in somatotypes are evident during adolescence, with boys more likely to show a greater prevalence of the mesomorphic–endomorphic component and girls primarily characterized by the endomorphic component [25]. Furthermore, mesomorphy develops more frequently in boys during puberty (as expected from the muscle growth), while girls become less mesomorphic and more endomorphic. This process takes 3 to 4 years and possibly longer in some individuals [16].

Sheldon et al. [26] argued that the somatotype is constant throughout life and is a genetically determined trait. It is now accepted that adult somatotypes can be influenced from adolescence to adulthood through physical activity or changes in diet, for example [11]. Changes in somatotypes with age have been associated with increasing health risks. The current study found an increase in the endomorphy component with age among adult females but not males, the mesomorphy component in both sexes, and a decreasing contribution of ectomorphy in males and females. The tendency for somatotypes to change with age is confirmed by, among others, cross-sectional studies of Canadians [21], Indus [22], and a Chuvasha population residing in a rural region of Central Russia [27]. In these studies, endomorphy increased over decades of life, and mesomorphy increased or remained the same over time.

Generally, the somatotypes distinguished in the present study significantly differentiated the basic anthropometric parameters in both sexes (BMI, WHR, waist circumference) known to be risk factors for health disorders associated with excessive fat mass. Indeed, endomorphy was associated with higher values of these measurements and ectomorphy with the lowest values. Consistent with these findings were the resting arterial blood pressure gradients, with endomorphs having the highest systolic and diastolic blood pressures and ectomorphs having the lowest (gradients for women reached statistically significant levels), which is in agreement with the results of other studies [28, 29]. The endomorphic body build and composition are common in adult populations of developed and developing countries, where excessive body weight is prevalent and often associated with a significant indicator of poorer health in both sexes [30, 31]. Overweight and obese individuals tend to show poorer cardiovascular parameters (including elevated systolic and diastolic blood pressure), metabolic disorders (e.g., type 2 diabetes), shortened life expectancy, and increased all-cause morbidity and mortality [30, 32].

Our results therefore emphasize that somatotypes differ significantly in variables widely recognized as predictors of civilization diseases. Waist circumference, for example, is a key predictor of type 2 diabetes and cardiovascular disease [33, 34], while BMI is a well-established risk factor for all-cause mortality [35]. The fact that endomorphs scored highest in these indices underlines the public health relevance of somatotyping as a complementary approach to traditional anthropometric screening.

A somatotype in itself does not determine health, though it can influence metabolism and susceptibility to certain diseases. Recent evidence also emphasizes that variability in somatotype reflects the combined effects of genetic and environmental factors rather than a direct causal determinant of health, which may explain some inconsistencies in the literature [36]. These findings should, however, be interpreted with caution. For example, Koleva et al. [37] reported that individuals with dominant mesomorphy and endomorphy, combined with low ectomorphy, had a higher prevalence of hypertension and liver disease, suggesting a predisposition rather than a direct causal effect. In contrast, a recent meta-analysis by Dou et al. [38] showed that higher somatotype scores in childhood were associated with a lower risk of postmenopausal breast cancer, highlighting paradoxical and age-dependent associations. Moreover, methodological factors may contribute to inconsistencies across studies. Bertuccioli et al. [39] demonstrated that somatotype estimation derived from bioimpedance analysis can differ substantially from traditional anthropometric approaches, affecting cross-study comparability. Finally, a recent scoping review of elite athletes by Martínez-Mireles et al. [40] emphasized the limited generalizability of somatotype data obtained in sports cohorts to community-based populations. Together, these findings underline both the potential and the limitations of somatotype assessment as a health-related marker.

Studies have shown that individuals with the highest endomorphy and mesomorphy and the lowest ectomorphy often suffer from hypertension and liver disease, while endomorphic mesomorphs most often experience gastrointestinal diseases, neuropathy, and lumbosacral radiculitis [37]. Endomorphy, which represents relative fatness, tends to be positively associated with risk factors such as systolic and diastolic blood pressure, fasting blood glucose, and blood lipids in older women, while ectomorphy, or relative linearity, tends to be inversely associated with risk factors in older men [41]. In addition, a comparison of the somatotypes of individuals representing the extremes of the distributions for each risk factor showed that those with higher risk profiles tended to be more endomorphic and mesomorphic and less ectomorphic than those with lower risk profiles [41]. Those with metabolic disorders such as type 2 diabetes are more likely to be endomorphic and have excess weight and body fat, particularly in the trunk [42]. Moreover, lean women with polycystic ovary syndrome (PCOS) were classified as mesomorphic endomorphs, in contrast to healthy women who were endomorphic mesomorphs [5]. Somatotype differentiated the respiratory parameters of adult males, with peripheral airway dysfunction observed more frequently in endomorphs and mesomorphs when compared to ectomorphs [43].

### Limitations

Potential limitations of the present study should be noted. One of the inclusion criteria was the good health of the participants and the absence of chronic disease symptoms. Although the participant’s health status was confirmed by a physician during the initial examination, it was not possible to exclude those with undiagnosed diseases such as hypertension or metabolic disorders. Nonetheless, it can be assumed that the observed relationship between somatotypes and body composition and cardiovascular parameters would not have changed if these potentially disease-burdened individuals had been excluded from the study. Next, study participants declared no or low levels of physical activity in their daily lives. A subjective assessment of activity levels may be prone to significant error. However, it is more likely that participants overestimated their activity rather than underestimated it [44], so the study group was unlikely to include athletes. In addition, the study group was deliberately heterogeneous, reflecting a typical adult urban population rather than a highly selected sample. This diversity strengthens the ecological validity of the findings while also limiting their generalizability to more specific subgroups.

There are also a few comments regarding the collected data. Resting systolic and diastolic blood pressure and heart rate were used to assess cardiovascular fitness. However, resting blood pressure measurements are insufficient for effectively monitoring and detecting possible abnormalities. Predicting the physical capacity of the participants in a simple exercise test would allow a more accurate estimation of the association between body build and cardiovascular risk. Moreover, the bone mass used in this study represents only an estimated value derived from BIA predictive equations and should not be interpreted as a direct measure of bone mineral content.

## Summary and conclusions

The results obtained for an adult population of men and women with low levels of physical activity or who declared themselves to be physically inactive showed significant sex differences in body build defined by somatotype. The mesomorphic and endomorphic components dominated to a similar extent in men, and the endomorphic component dominated in women, while the proportion of components depended on age. In general, the proportion of endomorphy and mesomorphy increased with age, while the proportion of ectomorphy significantly decreased with age.

Somatotyping significantly differentiated body composition in men and women. Endomorphs had the highest body fat component regardless of sex. The levels of the other body composition components (PMM, BM, FFM, and TBW) were highest in mesomorphs and lowest in ectomorphs. Furthermore, somatotype differentiated recognized cardiovascular risk factors, with endomorphs scoring worse for cardiovascular fitness and ectomorphs scoring best. The observed cardiovascular differences between body types reached statistical significance for adult women but not for men.

When assessing biological risk and predisposition to disease among physically low or inactive adults, somatotype may be a much better predictor than individual measurements, especially for women. Somatotype can be considered an appropriate tool for the comprehensive characterization of healthy and sick individuals. It provides compact, quantifiable, and comparable data and can be used to monitor the health status of the elderly population and plan nutritional intervention programs.

## Data Availability

All data generated or analysed during this study are included in this published article, any details are available from the corresponding author on reasonable request.

## Abbreviations

BIA: Bioelectrical Impedance Analysis
BM: Bone Mass
BMI: Body Mass Index
BP: Blood Pressure
FATP: Body Fat Percentage
FFM: Fat-Free Mass
GPAQ: Global Physical Activity Questionnaire
HWR: Height-Weight Ratio
IPAQ-SF: International Physical Activity Questionnaire – Short Form
MET: Metabolic Equivalent
SD: Standard Deviation
TMM: Total Muscle Mass
TBW: Total Body Water
WHR: Waist-to-Hip Ratio
